# miR-596 as a novel prognostic biomarker and tumor suppressor in breast cancer through targeting *EIF5AL1*

**DOI:** 10.1186/s41065-026-00641-6

**Published:** 2026-01-22

**Authors:** Rui Huang, Yan Jiang, Yan Bian, Dengyuan Zhong, Baoyong Lv

**Affiliations:** 1 Academic Theory Research Department, Mudanjiang Medical University, Mudanjiang, 157011 China; 2 One Departments of Cadre Ward, General Hospital of Southern Theater Command of Chinese PLA, Guangzhou, 510010 China; 3 Xi’an International University, Xi’an, 710077 China; 4 Department of Surgery II, The People’s Hospital of Shuangqiao Economic-Technological Development Zone, No. 6, Checheng Avenue, Chongqing, China 400900; 5 General Surgery, SHOUGUANG HOSPITAL OF T. C. M, No.3353, Shengcheng Street, Shouguang, 262700 China

**Keywords:** Breast cancer, MiR-596, EIF5AL1, Proliferation, Migration, Prognosis

## Abstract

**Background:**

In terms of global incidence and mortality, breast cancer continues to surpass all other cancers affecting women.

**Methods:**

To explore the role of miR-596, qRT-PCR was applied to measure its expression in tissue and cell samples from 137 enrolled subjects. The regulatory interaction between miR-596 and *EIF5AL1* was verified by dual-luciferase reporter assays. CCK-8 and Transwell assays were utilized to respectively measure the proliferation, migration, and invasion capabilities of the two breast cancer cell lines, MCF-7 and MDA-MB-231.

**Results:**

A significant downregulation of miR-596 was observed in breast cancer tissues and cell lines (all *P* < 0.001). Clinically, reduced miR-596 expression was associated with advanced TNM stage, lymph node metastasis, and inferior overall survival (*P* < 0.05). *EIF5AL1* was validated as a direct target gene of miR-596, and their expression levels were inversely correlated in clinical samples (*r* = -0.801, *P* < 0.001). Reintroduction of miR-596 markedly suppressed the proliferation, migration, and invasion of cancer cells, effects that were largely reversed by overexpressing *EIF5AL1* (all *P* < 0.001).

**Conclusion:**

In breast cancer, miR-596 suppresses malignancy and predicts prognosis by targeting *EIF5AL1*. Thus, therapeutic modulation of this axis offers novel avenues for treatment and risk assessment.

**Supplementary Information:**

The online version contains supplementary material available at 10.1186/s41065-026-00641-6.

## Introduction

Breast cancer continues to be the most frequently diagnosed cancer and represents the leading cause of cancer-related deaths in women worldwide, posing a substantial challenge to public health [1]. According to the International Agency for Research on Cancer, in 2022, this disease accounted for 23.8% of all incident cancers and 15.4% of female cancer fatalities, globally ranking fourth among all cancers in terms of mortality [2]. Although treatment strategies have improved considerably, the persistently high incidence and fatality rates of breast cancer continue to present considerable challenges to women’s health [3]. The frequent absence of obvious symptoms in early stages often leads to difficulties in early diagnosis, significantly affecting treatment efficacy and patient prognosis [4]. Therefore, in-depth research into its risk factors is of great importance for developing effective prevention strategies and reducing disease incidence.

Through seed sequence binding in the 3’UTRs of target mRNAs, miRNAs post-transcriptionally regulate gene expression by inducing mRNA degradation or translational repression [5]. Studies have shown that approximately 50% of miRNA genes are located in genomic fragile regions associated with tumorigenesis, where they are prone to deletion, amplification, or translocation, suggesting that miRNAs may play a key role in cancer development and progression [6]. For instance, in breast cancer, up to 73% of miRNA genes are located in genomic regions with copy number variations [7]. Accumulating evidence indicates that widespread dysregulation of miRNA expression drives the pathogenesis of numerous human diseases, most notably cancer.

miR-596 has been identified as a consistently downregulated tumor suppressor across multiple cancer types. For example, in non-small cell lung cancer, its low expression promotes brain metastasis, while in oral squamous cell carcinoma, it inhibits proliferation, metastasis, and apoptosis [8, 9]. Notably, miR-596 is also downregulated in breast cancer [10], yet its precise functional roles and underlying mechanisms remain poorly understood. This significant knowledge gap presents an opportunity to identify a novel biomarker with distinct advantages. Unlike some miRNAs with context-dependent functions, the conserved role of miR-596 across diverse malignancies suggests it governs a central oncogenic pathway, implying high clinical relevance and robust prognostic potential. Critically, our preliminary data point to *EIF5AL1* as a key downstream target, positioning the miR-596/*EIF5AL1* axis as a fundamentally new regulatory mechanism worthy of exploration.

This potential target, *EIF5AL1*, is itself a molecule of growing interest in tumor progression. In breast cancer, it has been identified as a key downstream target of the MKK3-MYC pathway and is markedly upregulated, potentially promoting disease progression through mechanisms like regulating EMT [11, 12]. However, the upstream regulation of *EIF5AL1*, particularly by miRNAs, remains largely unleftacterized, making its proposed control by miR-596 a pivotal and novel aspect of our study.

Based on this background, the present study seeks to evaluate the prognostic significance of miR-596 as a potential biomarker in breast cancer. Furthermore, we aim to explore its regulatory influence on critical cellular behaviors—including proliferation, migration, and invasion—through targeting *EIF5AL1*, in order to clarify its functional mechanism and involvement in breast cancer development.

## Materials and methods

### Study subjects

This study enrolled a total of 137 breast cancer patients from The People’s Hospital of Shuangqiao Economic-Technological Development Zone between 2018 and 2020. All experiments utilizing these clinical samples were performed with three independent biological replicates. The inclusion criteria for breast cancer patients were as follows: [1] diagnosis confirmed by histopathological examination; [2] good performance status (ECOG score 0–1 or Karnofsky score ≥ 70%); [3] absence of severe comorbidities, including hepatic, renal, pulmonary, or psychiatric diseases; [4] non-pregnant and non-lactating women; and [5] no history of other active malignancies within the past 5 years.

During the surgical procedures, paired specimens of tumor and corresponding normal tissues that fulfilled the established criteria were acquired. Each sample was reviewed independently by two pathologists to ensure diagnostic agreement. After labeling, the tissues were immediately frozen using liquid nitrogen and preserved at − 80 °C for subsequent use. A complete 60-month follow-up was conducted for all patients, with comprehensive survival records maintained. The experimental protocol received approval from the Ethics Committee of The People’s Hospital of Shuangqiao Economic-Technological Development Zone, and informed consent in written form was secured from every subject.

## Cell culture and transfection

The human mammary epithelial cell line MCF-10 A and four breast cancer cell lines (MCF-7, SK-BR-3, BT-549, MDA-MB-231), all sourced from Shanghai Tongwei Biotechnology, were cultured in high-glucose DMEM (Gibco, USA, catalog no. 11965118). The medium was supplemented with 10% fetal bovine serum (FBS, Gibco, USA, catalog no. E212000IC), 1% penicillin-streptomycin (Gibco, USA, catalog no. R01510), and all cells were grown at 37 °C under 5% CO₂.

When cells reached around 80% confluency during their logarithmic growth phase, they were collected for subsequent transfection procedures. Cells were transfected with Lipofectamine™ 3000 (Invitrogen, USA, catalog no. L3000150) for 6 h, and then incubated in fresh complete medium for another 48 h. Subsequently, total RNA was harvested to evaluate transfection efficiency and target gene expression changes by qRT-PCR. Additional phenotypic assays were subsequently carried out to validate these molecular findings.

## qRT-PCR

Total RNA was extracted from samples using TRIzol reagent (Invitrogen, USA, catalog no. 15596026CN), and qualified RNA was reverse-transcribed into cDNA using PrimeScript RT Master Mix (TaKaRa, Japan, catalog no. RR047A). Quantitative PCR was then performed using SYBR Premix Ex Taq (TaKaRa, Japan, catalog no. RR820A) on a QuantStudio 5 Real-Time PCR System (Applied Biosystems, USA). The thermal cycling protocol was as follows: initial denaturation at 95 °C for 30 s, followed by 38 cycles of 95 °C for 5 s and 60 °C for 30 s. GAPDH and U6 snRNA were used as endogenous controls for mRNA and miRNA, respectively. The 2^–ΔΔCt^ method was used for relative quantification. The sequences of all primers can be found in **Supplementary Table 1**. Each qRT-PCR reaction was performed with three technical replicates, and all experiments were conducted with three independent biological replicates (*n* = 3).

## Dual-Luciferase reporter assay

To experimentally confirm the potential targeting and regulatory interaction existing between miR-596 and the gene *EIF5AL1*, computational prediction tools were first employed to identify putative miR-596 binding sites located in the 3’UTR of *EIF5AL1*. Based on these predictions, two distinct types of luciferase reporter plasmids were subsequently designed and generated: one carrying the wild-type (wt) target sequence and another containing a corresponding mutant (mut) version of the binding site. These various recombinant plasmid constructs were then introduced via co-transfection into two human breast cancer cell lines, namely MCF-7 and MDA-MB-231, in combination with a series of different transfection groups. These experimental groups specifically included a blank control (Control), a mimic negative control (mimic NC), synthetic miR-596 mimic molecules, an inhibitor negative control (inhibitor NC), as well as a miR-596 inhibitor.

Forty-eight hours post-transfection, cell lysis was performed and luciferase activity was quantified with a dual-luciferase reporter assay kit (Solarbio, China, catalog no. E1960). Firefly luciferase signals were normalized to those of Renilla luciferase for intergroup comparison of relative activity. Each experimental condition was tested with four technical replicates, and the entire assay was independently repeated three times.

## Cell proliferation assay (CCK-8 Method)

Proliferation curves for MCF-7 and MDA-MB-231 cells were generated based on OD450 values obtained from CCK-8 assays (Dojindo Molecular Technologies, Japan, catalog no. CK04) performed at 0–72 h after transfection. The assay was performed with five technical replicates per group and independently repeated three times.

### Cell migration and invasion assay (Transwell Method)

Cell migration and invasion were assessed using Transwell assays (Corning, USA, catalog no. 354480) in MCF-7 and MDA-MB-231 cells under different transfection conditions (Control, mimic-NC, miR-596 mimic, miR-596 mimic + OE-NC, miR-596 mimic + OE-*EIF5AL1*). Cells that traversed the membrane after 24 h were stained and counted in five random fields per well. Each experimental group was set up in triplicate wells (technical replicates), and the entire experiment was repeated three independent times.

## Western blot analysis

Total protein was extracted from tissues and cell lines using RIPA lysis buffer (Beyotime, China, catalog no. P0013B) containing protease inhibitors and phosphatase inhibitors. The Bradford assay was then used to estimate protein concentrations. Protein samples were separated by SDS-PAGE and transferred to PVDF membranes (Thermo Fisher, USA, catalog no. PB9220). Membranes were blocked with 5% nonfat milk in Tris-Buffered Saline containing 0.05% Tween 20 (TBST) for 1.5 h and then incubated overnight at 4 °C with primary antibodies against *EIF5AL1* (Thermo Fisher, USA, catalog no. PA548316; dilution 1:1000) and GAPDH (Thermo Fisher, USA, catalog no. MA515738, dilution 1:2000). Following overnight incubation, the membranes were subjected to three TBST washes and then probed with HRP-conjugated secondary antibodies for 1.5 h at room temperature. The immunoreactive bands were subsequently detected employing an Enhanced Chemiluminescent Western Blotting Detection Reagent (GE Healthcare) and captured using a Chemiluminescent Imaging System (Tanon 5200). Each Western blot analysis was confirmed through three independent experimental repetitions.

### Data analysis

Data are expressed as mean ± standard deviation. Group differences, correlations, and survival rates were analyzed by t-test/ANOVA, Pearson’s method, and Kaplan-Meier curves, respectively, with multivariate Cox regression identifying prognostic factors (SPSS 23.0/GraphPad Prism 9; significance level: *P* < 0.05).

## Results

### Expression and prognostic significance of miR-596 in breast cancer

**Fig. 1:**
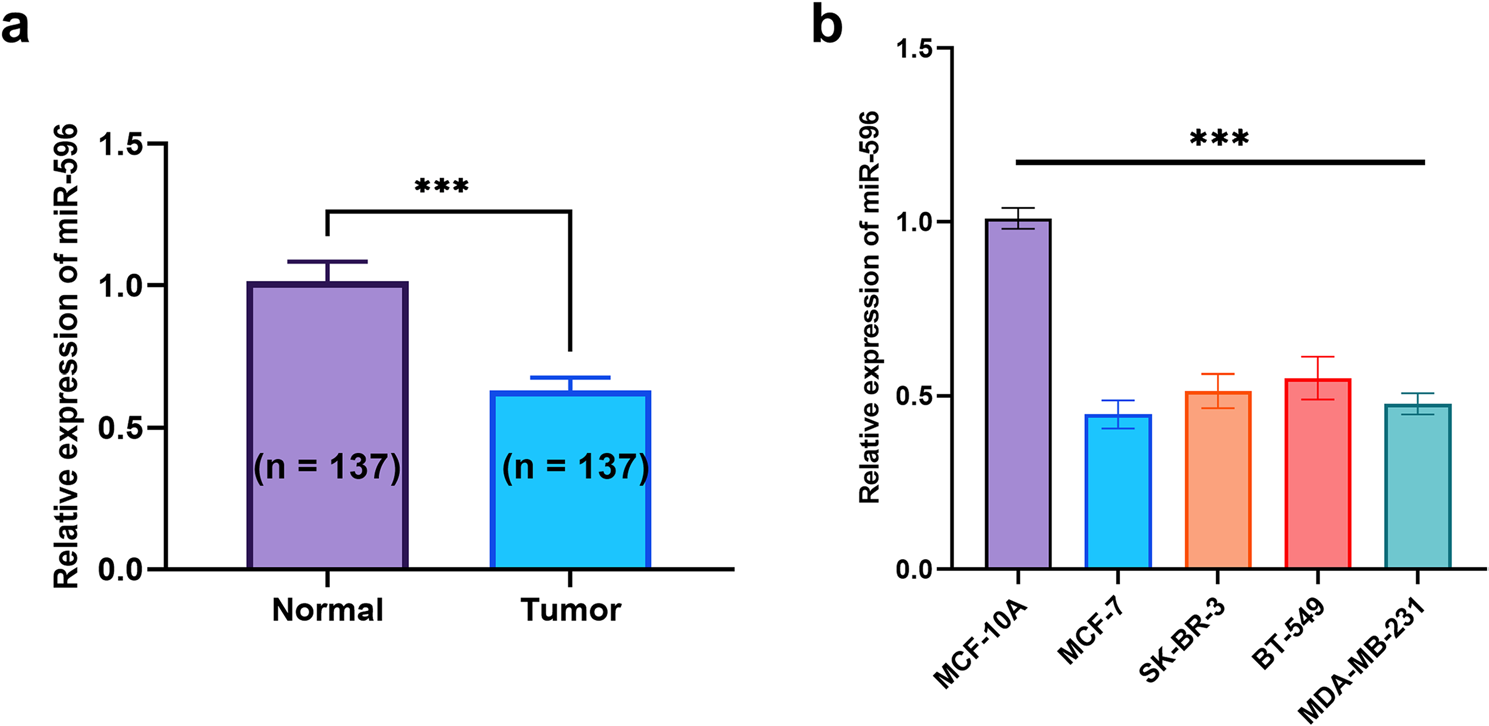
Expression of miR-596 in breast cancer tissues and cell lines. ***P < 0.001 (**a**) The relative expression level of miR-596 in breast cancer tissue compared to adjacent non-cancerous tissue (**b**) Relative expression of miR-596 in normal breast epithelial cell line MCF-10 A and breast cancer cell lines (MCF-7, SK-BR-3, BT-549, MDA-MB-231)

When compared with matched adjacent non-tumor tissue samples, breast cancer specimens showed a statistically significant reduction in the expression levels of miR-596 (*P* < 0.001, Fig. 1a). A similar trend was observed across several commonly used breast cancer cell lines—including MCF-7, SK-BR-3, BT-549, and MDA-MB-231—all of which displayed markedly decreased miR-596 expression relative to the normal mammary epithelial control cell line MCF-10 A (*P* < 0.001, Fig. 1b). These consistent findings suggest that the downregulation of miR-596 could represent a common occurrence during the progression of breast cancer.

**Fig. 2:**
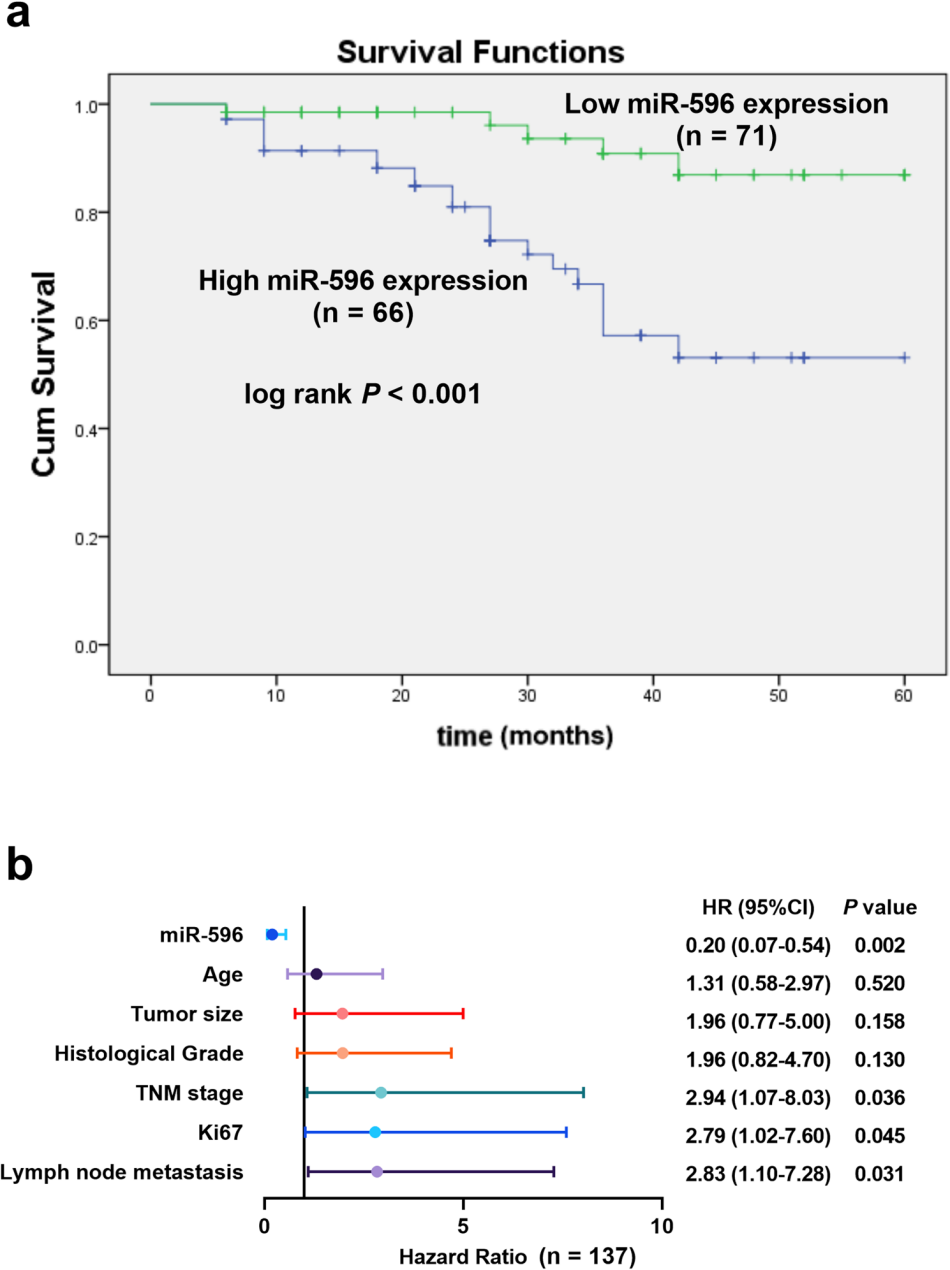
Prognostic value of miR-596 in breast cancer patients (**a**) Kaplan-Meier survival analysis of patients with high vs. low miR-596 expression (**b**) Multivariate Cox regression analysis of miR-596 and clinicopathological variables for overall survival

Based on the median expression level of miR-596, the cohort was stratified into two subgroups: a low-expression group (*n* = 71) and a high-expression group (*n* = 66). Statistical analysis revealed that reduced levels of miR-596 showed a significant correlation with more aggressive disease leftacteristics, including an advanced TNM stage (*P* = 0.018) and an increased incidence of lymph node metastasis (*P* = 0.031; Table 1). Importantly, the Kaplan–Meier survival curve demonstrated a markedly poorer overall survival for patients in the low-expression group compared to their high-expression counterparts (*P* < 0.001; Fig. 2a).

**Table 1:** The correlation between the expression of miR-596 and clinical leftacteristics

Parameters	Patients (*n* = 137)	miR-596 expression		*p*-value
		Low (*n* = 71)	High (*n* = 66)	
Age (years)				0.413
<50	82	45	37	
≥ 50	55	26	29	
Histological Grade				0.230
I-II	80	38	42	
III	57	33	24	
Tumor size (cm)				0.262
<2	76	36	40	
≥ 2	61	35	26	
TNM stage				0.018
Ⅰ-Ⅱ	75	32	43	
Ⅲ-Ⅳ	62	39	23	
Lymph node metastasis				0.031
no	72	31	41	
yes	65	40	25	
Ki67				0.236
< 20%	64	28	36	
≥ 20%	73	43	30	
Molecular subtype				
Luminal A	53	31	22	0.296
Luminal B	29	15	14	
HER2-enriched	21	12	9	
Triple-negative	34	13	21	

*TNM stage* Tumor, Node, Metastasis Staging System; A *p*-value < 0.05 indicates a significant difference

Furthermore, to determine independent prognostic factors, a multivariate Cox regression analysis was performed. The results confirmed that low miR-596 expression (HR = 0.20, 95% CI: 0.07–0.54, *P* = 0.002), along with advanced TNM stage, high Ki67 expression, and the presence of lymph node metastasis, stood out as significant independent predictors of poor prognosis (Fig. 2b). In summary, these findings consistently indicate that decreased miR-596 expression could potentially function as a valuable biomarker for assessing disease progression and predicting clinical outcomes in breast cancer.

### Validation of the targeting regulatory relationship between miR-596 and EIF5AL1

**Fig. 3:**
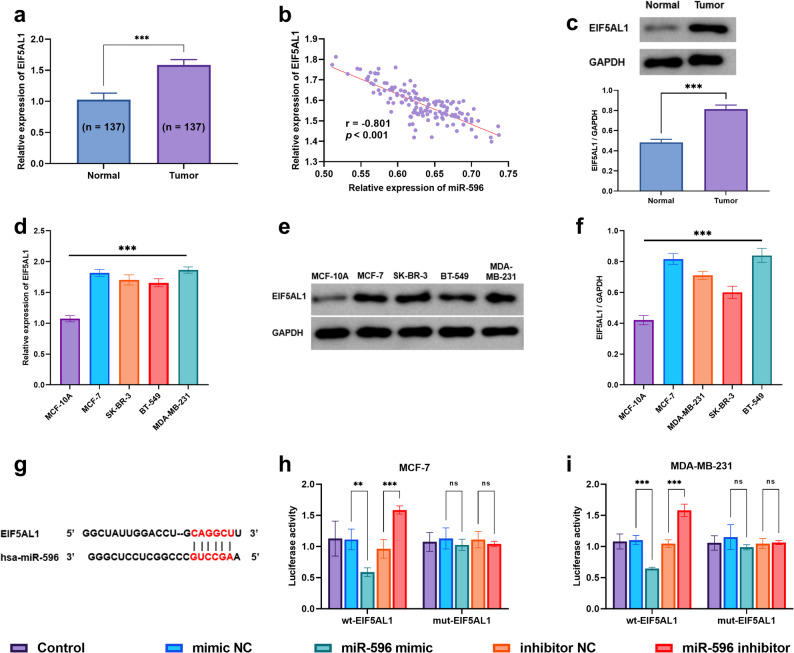
*EIF5AL1* is a direct target of miR-596 in breast cancer. **P < 0.01, ***P < 0.001, ns: *P* > 0.05 (**a**) The relative expression level of *EIF5AL1* in breast cancer tissue compared to adjacent non-cancerous tissue (**b**) Correlation analysis between miR-596 and *EIF5AL1* expression (**c**) Western blot analysis of *EIF5AL1* protein expression in normal and breast cancer tissues, and its quantitative statistical results (**d**) Relative expression of *EIF5AL1* in normal breast epithelial cells (MCF-10 A) and breast cancer cell lines (**e-f**) Western blot analysis of *EIF5AL1* protein expression in normal mammary epithelial cells (MCF-10 A) and breast cancer cell lines (**e**), and the corresponding quantitative statistical results (**f**) (**g**) Predicted binding site of miR-596 within the 3′UTR of *EIF5AL1* (h–i) Luciferase reporter assay showing activity in cells co-transfected with miR-596 mimic and wild-type (wt) or mutant (mut) *EIF5AL1* 3′UTR reporter constructs

When compared with surrounding non-cancerous tissue samples, the expression of *EIF5AL1* was found to be substantially upregulated within breast cancer specimens, a difference that was highly statistically significant (*P* < 0.001, as shown in Fig. 3a). An analysis examining the correlation between the expression profiles of miR-596 and *EIF5AL1* among individuals diagnosed with breast cancer (Fig. 3b) further demonstrated a strong and significant inverse relationship (with a correlation coefficient *r* = −0.801, *P* < 0.001). To further confirm the dysregulation of *EIF5AL1* in breast cancer, we detected its expression at the protein level: Western blot analysis showed that *EIF5AL1* protein abundance was also markedly increased in breast cancer tissues relative to adjacent normal tissues, which was consistent with the mRNA-level changes and exhibited high statistical significance (*P* < 0.001, Fig. 3c).

At the cellular level, evaluations across several commonly studied breast cancer cell lines—including MCF-7, SK-BR-3, BT-549, and MDA-MB-231—showed that *EIF5AL1* was expressed at markedly elevated levels relative to its expression in the normal mammary epithelial control cell line MCF-10 A (*P* < 0.001, Fig. 3d). We further validated this trend at the protein level: Western blot analysis (Fig. 3e) and quantitative statistical results (Fig. 3f) consistently demonstrated that *EIF5AL1* protein expression was significantly upregulated in the above breast cancer cell lines compared with MCF-10 A, with the difference reaching high statistical significance (*P* < 0.001). These collective observations suggest that *EIF5AL1* could potentially play an oncogenic role in the development and progression of breast cancer.

To further strengthen this finding with independent data, we analyzed the expression of *EIF5AL1* in the large public TCGA-BRCA cohort. Consistent with our own observations, *EIF5AL1* mRNA was significantly upregulated in breast tumor tissues compared to normal adjacent tissues. Kaplan‑Meier survival analysis further revealed that high expression of *EIF5AL1* was associated with a poorer prognosis trend (Supplementary Fig. 1).

Bioinformatic analysis indicated that complementary binding sequences exist between hsa-miR-596 and the 3′UTR of *EIF5AL1*, suggesting that *EIF5AL1* is a potential target gene of miR-596 (Fig. 3g). A dual-luciferase reporter assay provided further validation for this predicted binding interaction (Fig. 3h–i). In both MCF-7 and MDA-MB-231 cells, co-transfection with miR-596 mimic and the wild-type reporter vector (wt-*EIF5AL1*) significantly suppressed luciferase activity (*P* < 0.01), while co-transfection with miR-596 mimic and the mutant reporter vector (mut-*EIF5AL1*) had no significant effect. Conversely, transfection with miR-596 inhibitor significantly enhanced the luciferase activity of the wt-*EIF5AL1* reporter vector (*P* < 0.001). Collectively, these findings demonstrate that miR-596 directly targets the 3’UTR of *EIF5AL1* to suppress its expression.

### miR-596 regulates breast cancer cell proliferation and migration by targeting EIF5AL1 for suppression

**Fig. 4:**
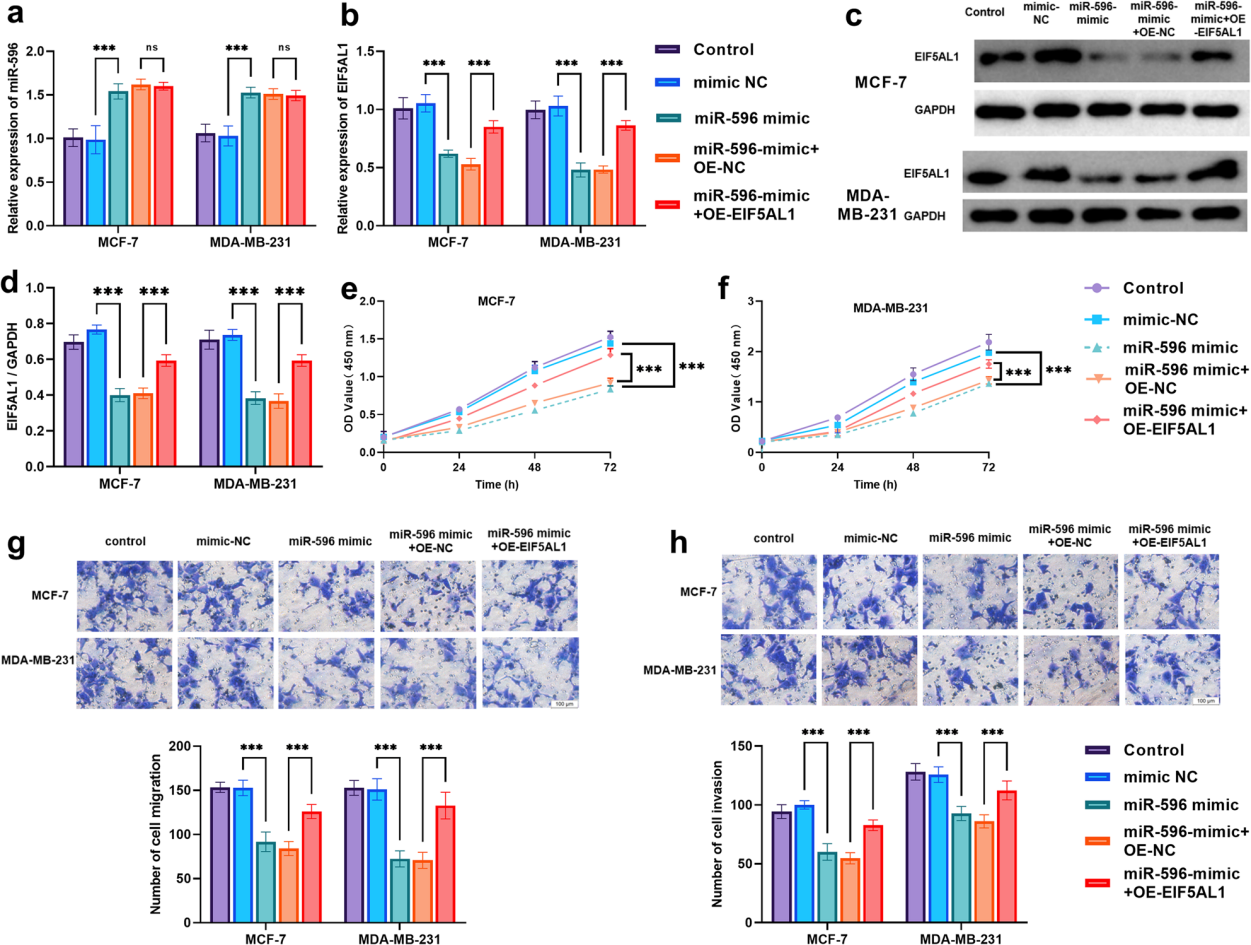
Overexpression of *EIF5AL1* reverses the tumor-suppressive effects of miR-596 in breast cancer cells. ***P < 0.001 (**a–b**) Relative expression of miR-596 (**a**) and *EIF5AL1* (**b**) in cells transfected with miR-596 mimic alone or in combination with *EIF5AL1* overexpression plasmid (OE-*EIF5AL1*) or its empty vector control (OE-NC) (**c–d**) Western blot analysis of *EIF5AL1* protein expression (**c**) and its quantitative statistical results (**d**) in cells treated with control, miR-596 mimic, miR-596 mimic + OE-NC, or miR-596 mimic + OE-*EIF5AL1* (e–f) Proliferation ability measured by CCK-8 assay in MCF-7 (**e**) and MDA-MB-231 (**f**) cells under different treatment conditions (**g–h**) Cell migration (**g**) and invasion (**h**) assays showing functional rescue following co-transfection with miR-596 mimic and OE-*EIF5AL1*

As clearly demonstrated in Fig. 4a, the process of transfection using a synthetic miR-596 mimic resulted in a substantial and statistically significant upregulation in the expression levels of miR-596 within both MCF-7 and MDA-MB-231 human breast cancer cell lines (*P* < 0.001). In contrast, when miR-596 mimic was co-transfected with an *EIF5AL1* overexpression vector, no significant change in miR-596 expression was observed. Similarly, as illustrated in Fig. 4b, transfection with miR-596 mimic markedly suppressed *EIF5AL1* expression in both cell lines (*P* < 0.001), while co-transfection with the *EIF5AL1* overexpression vector significantly restored its expression level. This regulatory effect was further validated at the protein level: Western blot (Fig. 4c) and quantitative analysis (Fig. 4d) showed that miR-596 mimic reduced *EIF5AL1* protein levels, which was reversed by *EIF5AL1* overexpression (*P* < 0.001). These results further confirm that *EIF5AL1* can be regulated as a target gene of miR-596 and demonstrate the reliability and rescuing effect of the experimental system used in this study.

In terms of cell proliferation, transfection with miR-596 mimic significantly inhibited the proliferative activity of breast cancer cells (MCF-7 and MDA-MB-231) (*P* < 0.001). However, when miR-596 mimic was co-transfected with OE-*EIF5AL1*, cellular proliferation ability was notably restored compared to the miR-596 mimic + OE-NC group (Fig. 4e–f), indicating that *EIF5AL1* overexpression partially reversed the suppressive effect of miR-596 on breast cancer cell proliferation.

As demonstrated by the migration (Fig. 4g) and invasion (Fig. 4h) assays, transfection with miR-596 mimic significantly reduced the number of migrating and invading MCF-7 and MDA-MB-231 cells compared to the mimic-NC group (all *P* < 0.001). Conversely, co-transfection with miR-596 mimic and OE-*EIF5AL1* led to a significant recovery in cell migration and invasion capabilities compared to the miR-596 mimic + OE-NC group (all *P* < 0.001). These findings indicate that *EIF5AL1* overexpression partially reverses the inhibitory effects of miR-596 on breast cancer cell migration and invasion.

## Discussion

Breast cancer remains a leading cause of cancer-related morbidity and mortality in women worldwide, with its high heterogeneity posing significant challenges for prognosis and treatment [13]. While current clinical practice relies on conventional indicators like TNM stage and Ki-67, microRNAs have emerged as promising molecular markers. For instance, miR-205-5p is frequently downregulated [14, 15], whereas miR-155 is often upregulated in breast cancer [16]. Several miRNAs, including miR-589, miR-10b, and miR-373, have also demonstrated diagnostic potential [17–19]. Nevertheless, the independent prognostic value of these markers requires further validation.

This study systematically elucidates the role of the miR-596/*EIF5AL1* regulatory axis in breast cancer progression through comprehensive analysis of its expression patterns, clinical significance, and molecular mechanisms. Compared with existing research, our findings not only provide a novel biomarker for prognostic assessment but, more importantly, reveal a new regulatory mechanism distinct from traditional signaling pathways at the molecular level.

Compared with currently known breast cancer-associated miRNAs, miR-596 demonstrates multiple unique advantages. First, in terms of functional specificity, unlike the oncogenic and functionally complex miR-155, miR-596 serves as a clear tumor suppressor whose expression level directly correlates with the loss of tumor suppressive function, providing a more solid theoretical foundation for its clinical application. Second, regarding diagnostic efficacy, although miR-589 shows comparable diagnostic accuracy (AUC = 0.85) [19], miR-596 functions through targeting *EIF5AL1*, a non-canonical translation factor, which represents a mechanism distinctly different from miR-205-5p that targets traditional signaling pathway molecules like LRP6 [20]. This mechanistic innovation endows miR-596 with unique value in prognostic assessment and therapeutic targeting of breast cancer.

To enhance the reliability and clinical applicability of our results, we performed external validation of the expression pattern and prognostic significance of *EIF5AL1* using the TCGA-BRCA database. The analysis results were highly consistent with our experimental findings: *EIF5AL1* was significantly upregulated in breast cancer tissues.

In terms of mechanism investigation, we validated the direct targeting relationship between miR-596 and *EIF5AL1* through multi-level experiments. Results from bioinformatic prediction, expression correlation analysis, and dual-luciferase reporter assays collectively confirmed that miR-596 negatively regulates *EIF5AL1* expression by specifically binding to its 3’UTR region [21–23]. It is noteworthy that although EIF5A family members share high sequence homology, they exhibit fundamental differences in post-translational modifications [21, 24, 25]. The unique leftacteristics of *EIF5AL1*—its inability to undergo hypusination and rapid degradation via the proteasomal pathway—suggest it may serve as a critical node in tumor proteome regulation. This discovery not only expands our understanding of breast cancer molecular mechanisms but also provides direction for developing new therapeutic strategies.

At the functional level, we confirmed that miR-596 overexpression significantly inhibits the proliferation, migration, and invasion capabilities of breast cancer cells, and these phenotypic effects were markedly reversed upon *EIF5AL1* restoration. This finding directly demonstrates that *EIF5AL1* is the key downstream effector molecule mediating the tumor-suppressive function of miR-596 [26, 27]. Compared to conventional mechanisms such as miR-205-5p targeting *LRP6* [20] or miR-145-5p targeting *ARF6* [28], the mechanism by which miR-596 influences tumor progression through regulating the translation elongation factor *EIF5AL1* is more innovative, potentially offering new avenues for breast cancer treatment.

Despite these findings, several limitations should be acknowledged. First, the clinical sample size, though statistically informative, remains limited to a single institution, which necessitates further validation through larger multi-center cohorts to enhance the generalizability of our conclusions. More importantly, the present study primarily relies on in vitro cellular experiments to establish the regulatory axis between miR-596 and *EIF5AL1*. The absence of in vivo data from animal models, such as xenograft experiments, leaves the physiological relevance and therapeutic potential of this axis in a complex tumor microenvironment insufficiently validated. This gap somewhat limits the translational impact of our findings. Furthermore, the precise molecular mechanisms downstream of *EIF5AL1*, including its potential involvement in classic oncogenic pathways like Wnt/β-catenin or PI3K/AKT, remain unclear. Future investigations should prioritize employing in vivo models to evaluate the anti-tumor efficacy of miR-596 agomir or *EIF5AL1*-specific inhibitors, conducting integrated omics analyses to delineate the comprehensive network regulated by *EIF5AL1*, and exploring its clinical applicability, such as developing miR-596-based therapeutic strategies or combination treatment regimens.

Based on current research findings, future research directions should include: establishing animal models to evaluate the therapeutic potential of targeting the miR-596/*EIF5AL1* axis; employing omics technologies to comprehensively analyze the molecular network regulated by *EIF5AL1*; and exploring therapeutic strategies based on miR-596 mimics or *EIF5AL1* inhibitors. These investigations will not only contribute to a deeper understanding of breast cancer pathogenesis but may also provide new targets and strategies for clinical treatment.

## Conclusion

This study reveals that miR-596 is markedly downregulated in both breast cancer tissues and cell lines, with its reduced expression associated with advanced TNM stage, lymph node metastasis, and unfavorable patient outcomes. Functioning as an independent prognostic indicator, miR-596 exhibits tumor-suppressive properties and represents a potential biomarker. Through bioinformatic analysis, dual-luciferase reporter assays, and functional rescue studies, *EIF5AL1* was identified as a direct target of miR-596. Further experiments confirmed that miR-596 suppresses the proliferation, migration, and invasion of breast cancer cells by regulating *EIF5AL1*, uncovering a previously unrecogned regulatory pathway in breast cancer development.

## Supplementary Information

Supplementary Material 1.

## Data Availability

Further enquiries can be directed to the corresponding author.
