# Supplementary material for: miR-596 as a novel prognostic biomarker and tumor suppressor in breast cancer through targeting EIF5AL1
Source: Hereditas. 2026 Jan 22;163:28. doi: 10.1186/s41065-026-00641-6 (PMC12911248; doi:10.1186/s41065-026-00641-6)
Supplement: Supplementary file 1 — Supplementary Material 1. [file 41065_2026_641_MOESM1_ESM.docx]

Supplementary Table 1. Primer sequences for real-time quantitative polymerase chain reaction (RT-qPCR).

| Gene |  | Sequences (5´-3´) |
| --- | --- | --- |
| miR-596 | Forward | AAGCCTGCCCGGCTCCT |
|  | Reverse | GCTGTCAACGATACGCTACGT |
| EIF5AL1 | Forward | GAATTCGCCACCATGGCAGAT |
|  | Reverse | TTGCCATGGCCTTGATTGCAA |
| U6 | Forward | CTCGCTTCGGCA GCACA |
|  | Reverse | AACGCTTCAC GAATTTGCGT |
| GAPDH | Forward | TCGGAGTCAACGGATTTGGT |
|  | Reverse | TTCCCGTTCTCAGCCTTGAC |
